# Soluble Guanylate Cyclase Stimulator Vericiguat Enhances Long-Term Memory in Rats without Altering Cerebral Blood Volume

**DOI:** 10.3390/biomedicines9081047

**Published:** 2021-08-19

**Authors:** Ellis Nelissen, Elentina K. Argyrousi, Nick P. Van Goethem, Fuqiang Zhao, Catherine D. G. Hines, Gayathri Swaminath, Michael Gerisch, Joerg Hueser, Peter Sandner, Jos Prickaerts

**Affiliations:** 1 Department of Psychiatry and Neuropsychology, School for Mental Health and Neuroscience (MHeNS), Maastricht University, Universiteitssingel 50, 6229 ER Maastricht, The Netherlands; e.nelissen@maastrichtuniversity.nl (E.N.); ea2693@cumc.columbia.edu (E.K.A.); n.vangoethem@maastrichtuniversity.nl (N.P.V.G.); 2 Merck & Co., Inc., Kenilworth, NJ 07033, USA; fzhao625@gmail.com (F.Z.); catherine.hines@merck.com (C.D.G.H.); 3 Merck & Co., Inc., South San Francisco, CA 94080, USA; gayathri.swaminath@merck.com; 4 Bayer AG, Pharmaceuticals R&D, Pharma Research Center, 42113 Wuppertal, Germany; michael.gerisch@bayer.com (M.G.); joerg.hueser@bayer.com (J.H.); peter.sandner@bayer.com (P.S.); 5 Hannover Medical School, Institute for Pharmacology, 30625 Hannover, Germany

**Keywords:** soluble guanylate cyclase, cGMP, AMPA, acquisition, memory

## Abstract

Vascular cognitive impairment (VCI) is characterized by impairments in cerebral blood flow (CBF), endothelial function and blood–brain barrier (BBB) integrity. These processes are all physiologically regulated by the nitric oxide (NO)-soluble guanylate cyclase (sGC)-cGMP signaling pathway. Additionally, cGMP signaling plays an important role in long-term potentiation (LTP) underlying memory formation. Therefore, targeting the NO-sGC-cGMP pathway may be a therapeutic strategy for treating VCI. Hence, in this study we investigated whether sGC stimulator vericiguat has potential as a cognitive enhancer. The effects of vericiguat on long-term memory were measured in rats using an object location task. Due to the low brain-penetrance of vericiguat found in this study, it was investigated whether in the absence of BBB limitations, vericiguat enhanced hippocampal plasticity using an ex vivo memory acquisition-like chemical LTP model. Finally, peripheral effects were measured by means of blood pressure and cerebral blood volume. Vericiguat successfully enhanced long-term memory and increased hippocampal plasticity via enhanced translocation of α-amino-3-hydroxy-5-methyl-4-isoxazole propionic acid (AMPA) receptors to the cell membrane, while blood pressure and cerebral blood volume were unaltered. Although the memory enhancing effects in this study are likely due to peripheral effects on the cerebral microvasculature, sGC stimulation may provide a new therapeutic strategy for treating VCI, especially when BBB integrity is reduced.

## 1. Introduction

Vascular cognitive impairment (VCI) is a broad term that covers a large group of neurological and neurodegenerative disorders that are all characterized by cognitive alterations caused by a vascular component [1]. The pathobiology involves reduced cerebral blood flow (CBF), inflammation, white matter lesions, endothelial dysfunction, and blood–brain barrier (BBB) leakage (for a review see [2]). Under physiological conditions, nitric oxide (NO) is one of the key regulators of most of these processes. NO regulates CBF, for example by controlling the tone of vascular smooth muscle cells (VSMCs). In this process, NO produced by endothelial nitric oxide synthase (eNOS) diffuses to VSMCs and can stimulate the enzyme soluble guanylate cyclase (sGC) to produce cGMP. This NO-sGC-cGMP cascade can trigger intracellular processes in VSMCs to eventually induce VMSC relaxation and vasodilation [3,4,5]. This same NO-sGC-cGMP signaling pathway can also regulate endothelial cell permeability and neurovascular coupling, and via the phosphorylation of vasodilator-stimulated phosphoprotein (VASP) this pathway can also regulate tight junction formation in the BBB [6,7,8,9,10].

Memory is the process in which acquired information is stored and retained over time so it can be retrieved when required [11]. The information is stored and maintained by the constant modification of cells, their synapses, and the synaptic connections between cells, which can be referred to as cellular or brain plasticity and synaptic plasticity [12]. It is thought that long-term potentiation (LTP) is the underlying mechanism behind cellular and synaptic plasticity, forming the molecular basis of memory [13,14,15,16]. Research has shown that LTP relies, at least in part, on the dynamics of α-amino-3-hydroxy-5-methyl-4-isoxazole propionic acid (AMPA) receptors [17,18]. AMPA receptors (AMPARs) are tetrameric ionotropic glutamatergic receptors that mostly exist as either homomers or heteromers of the isoforms of four distinct subunits: GluA1, GluA2, GluA3 and GluA4 (formerly known as GluR1-4) [19]. It is thought that GluA1/1 homomers and GluA1/2 heteromers are mostly responsible for synaptic strengthening in LTP [20], and the rapid membrane insertion of GluA1 specifically is thought to underly initial memory processes [18,21,22]. NO-sGC-cGMP signaling is also known to underly memory formation and LTP ([23,24]), and cGMP is able to induce the phosphorylation of the carboxy tail of GluA1 on S845 via protein kinase G (PKG), which is thought to be an important drive for GluA1 mobilization to the membrane [25,26]. Indeed, enhancing neuronal cGMP signaling has recently been shown to induce GluA1 mobilization to the membrane in the hippocampus of mice [21].

Cyclic nucleotides (i.e., cGMP and cAMP) are broken down by cyclic nucleotide phosphodiesterases (PDEs), and many studies have shown that inhibition of PDEs can enhance cyclic nucleotide signaling and thereby enhance cognition [27,28]. There are 11 subclasses of PDEs of which PDE4, 7 and 8 are cAMP-specific, PDE5, 6 and 9 cGMP-specific, while the others act on both cAMP and cGMP [29,30]. Of the cGMP-specific PDEs, PDE5 has been extensively researched for its potential as a target for cognitive enhancement through the use of PDE5 inhibition [27,31,32]. PDE5 inhibitors are well-known for their treatment of erectile dysfunction, e.g., sildenafil and vardenafil [33]. However, both sildenafil and vardenafil have also been extensively researched for their cognition enhancing properties [21,31,32]. Unfortunately, vardenafil showed no effect on cognitive performance in humans [34], and mRNA expression of PDE5 in adult rat brains was found to be mainly restricted to the cerebellum [35]. In addition, the efficacy of PDE5 and PDE9 inhibitors is critically dependent on endogenous NO and, consequently, cGMP production, since they can inhibit cGMP degradation only. Novel pharmacological interventions have therefore focused upstream of cGMP to enhance its signaling, namely by enhancing cGMP production. More recently, sGC stimulators have been identified which are able to stimulate sGC even in the absence of NO and potently enhance cGMP production and cGMP downstream signaling. The novel sGC stimulators were so far mostly profiled in cardiovascular and cardiopulmonary diseases. For example, riociguat, the first in class sGC stimulator, was approved for the treatment of pulmonary arterial hypertension (PAH) and chronic thromboembolic pulmonary hypertension (CTEPH) in 2013, and it has been shown pre-clinically to enhance memory performance in mice via peripheral mechanisms [36]. One of the most recent clinically investigated sGC stimulators for cardiovascular disease is the long-acting sGC stimulator vericiguat [37,38,39,40]. Additionally, a pivotal phase 3 clinical trial with vericiguat for the treatment of heart failure (HFrEF) has successfully been completed [41]. Since there is an overlap of cardiovascular diseases and heart failure with memory deficits and dementias [42,43] and NO-sGC-cGMP activity is a key mediator in many of the physiological processes that become pathological in VCI, it would be highly interesting to study potential benefits of vericiguat on cognitive dysfunction.

Since the potential cognition enhancing effects of vericiguat have yet to be explored, this preclinical study aims to investigate for the first time the effects of vericiguat on memory acquisition in vivo in an object location task (OLT) paradigm for spatial memory in rats. The ability of vericiguat to cross the BBB is also investigated. Since riociguat showed memory enhancement via peripheral effects due to a lack of brain penetrance in a similar study [36], in this study with vericiguat the hippocampal plasticity effects of vericiguat are investigated in an ex vivo acquisition-like chemical LTP model by measuring the effects on GluA1-containg AMPA receptor dynamics. This ex vivo model is meant to mimic the effects of vericiguat on hippocampal memory processes in the absence of BBB limitations, to identify vericiguat’s potential for central effects under leaky BBB circumstances. The effects of vericiguat on blood pressure in rats are also measured to examine the therapeutic window for peripheral side effects. Further, the dilation effect of vericiguat on the cerebrovasculature is measured by cerebral blood volume MRI.

## 2. Methods

### 2.1. Animals

Overall, the main goal was to show a robust effect of vericiguat on memory performance, excluding peripheral side effects such as effects on the mean arterial pressure. For this, we used the most optimal species/strain/sex available to us for each technique. This study was therefore explorative without any clear prior indication of effectiveness and was set up accordingly. All experimental procedures were approved by the local ethical committee of Maastricht University for behavioral animal experiments (approval code: DEC 2012-063; approval date: 3 November 2016; Licensed animal ethical committee Ministry of VWS, GZBIVVB981845) and by the regional German regulatory authority (approval code: 400a14, N0400a0207; approval date: 14 September 2017; German regulatory authority for animal experiments LANUV NRW), and the institutional animal care and use committee of Bayer AG for blood-pressure and PK measurements met governmental guidelines. All studies are reported in concordance with ARRIVE guidelines [44]. For the behavioral study, twenty-four 3–4-month-old male Wistar rats (Charles River, Sulzfeld, Germany) were used (average body weight at the beginning of the study: 292 g). The animals were housed individually in a standard tecniplast greenline IVC cage system on sawdust bedding in an air-conditioned room (about 20 °C). They were kept under a reversed 12/12 h light/dark cycle (lights on from 19:00 to 7:00) and had free access to food and water. Rats were housed and tested in the same room. A radio, which was playing softly, provided background noise in the room. All testing was carried out between 09:00 and 17:00. The rat strain was chosen based on its suitability for the behavioral task in this study [45]. For object recognition testing, historical research performed by us is most optimized for rats; therefore, rats are preferred, although the outcome between rats and mice is similar [45,46,47].

For the chemical LTP study, sixteen 2–3-month-old male C57BL/6 mice (Charles River, Sulzfeld, Germany) were used (average body weight at the beginning of the study: 24.4 g). The animals were housed socially in groups of 4 in a standard tecniplast blueline IVC cage system on sawdust bedding in an air-conditioned room (about 20 °C). They were kept under a normal 12/12 h light/dark cycle (lights on from 7:00 to 19:00) and had free access to food and water. Mice were specifically chosen based on the smaller size of hippocampal slices compared to rats, since rat slices are technically not compatible with the experimental set-up and mice are preferred. Larger (rat) slices could result in overlap between slices in the incubation chambers, and this can influence slice recovery and responsiveness.

Cerebral blood volume animal studies were approved by the Institutional Animal Care and Use Committee (IACUC) of Merck Sharp & Dohme, Inc. located in West Point, PA, USA (approval code: 2019-600987-AUG; approval date: 18 August 2017). A total of 35 female Sprague Dawley rats of 8–12 weeks old and 170–250 g (Taconic Biosciences, Germantown, NY, USA) were pair housed under normal 12/12 h light/dark cycles and provided food and water ad libitum. The female Sprague Dawley rats were specifically chosen due to the consideration of body size, as the cradle for magnetic resonance imaging (MRI) was designed for smaller rats (<400 g), while male rats are generally >400 g. Clinically vericiguat has been approved for the treatment of heart disease for both male and female patients [48], and it has previously been shown that the differences between sexes do not affect the brain:body mass ratio [49] while cerebral perfusion rates of male and female rats are nearly identical (0.48 and 0.51 mL/g/min respectively). Therefore, we did not anticipate a sex-dependent effect of vericiguat on cerebral blood volume.

### 2.2. Materials

Vericiguat was supplied by Bayer AG. Donepezil, an acetylcholinesterase inhibitor (AChEI), was generously donated by Abbott (Weesp, The Netherlands). Methyl 2-hydroxyethyl cellulose (Tylose^®^ MH300, cat# 93800, lot# 0001404713) and Tween-80 (polyoxyethylenesorbitan monooleate, cat# P8074, lot# BCBF2368V) were purchased from Sigma-Aldrich Chemie bv (Steinheim, Germany). Saline (natriumchloride 0.9%, Chargenr: 151548082) was purchased from B. Braun Melsungen AG (Melsungen, Germany). Forskolin was purchased from Tocris Bioscience (#1099, Abingdon, UK), rolipram was purchased from Abcam (#ab120029, Cambridge, UK), and sulfo-NHS-SS-biotin (#21328) and streptavidin-coated Dynabeads (#65601) were purchased from Thermo Scientific (Bleiswijk, The Netherlands).

### 2.3. Treatment

All test compounds were freshly prepared on each experimental day. Vericiguat was dissolved in 0.5% Tylose solution (98% of the end volume) with 2% Tween-80, whereas donepezil was dissolved in saline.

Vericiguat was tested at doses of 0.03, 0.1, 0.3, 1.0 and 3.0 mg/kg, and donepezil was tested at the active dose of 1.0 mg/kg as a positive control for memory enhancement. The compounds were tested in a time-dependent memory deficit model, i.e., a 24 h inter-trial interval OLT. All compounds were administered p.o. at 2 mL/kg 30 min before T1 to investigate the effects on the memory acquisition process. The order of the treatments was balanced and randomized to prevent the data from being distorted by potential object- and side-preferences of the animals. The experimenter was always blinded to the conditions during the experiment.

For the cerebral blood volume measurements, vericiguat was sonicated and dissolved in PEG400 and administered subcutaneously (s.c) at different doses of 0.03 (n = 4), 0.1 (n = 7), 0.3 (n = 7) and 1 (n = 5) mg/kg subcutaneously as a bolus. A total of 10 rats were administered PEG400 alone as vehicle. A quantity of 8 mg/mL sildenafil (Viagra ^®^, Pfizer Inc, New York, NY, USA) was dissolved in saline (0.8 mg/mL) and administered at a dose of 9 mg/kg intraperitoneally as a positive control (n = 2). Vehicle test with saline was performed before sildenafil treatment.

### 2.4. Object Location Task

The object location task (OLT) is a one-trial learning task that assesses spatial memory, and it is derived from the object recognition test (ORT) first described by Ennaceur and Delacour [50]. The OLT was performed as described elsewhere [51,52]. In brief, in the first trial, the rat was placed into a circular arena with two identical objects (learning trial, T1). After a delay, the rat was placed back into the arena with the same identical objects, but one object is displaced (testing trial, T2). By changing the spatial arrangement, it is measured whether the rat remembers the old spatial arrangement. The duration of each trial was 3 min. The total time an animal spent exploring each object during T1 and T2 was recorded manually with a personal computer.

The apparatus consisted of a circular arena, 83 cm in diameter. The back-half of the 40 cm-high arena wall was made of gray polyvinyl chloride, and the front-half consisted of transparent polyvinyl chloride. The light intensity was equal in the different parts of the apparatus, as fluorescent red tubes provided a constant illumination of about 20 lux on the floor of the apparatus. In the first (learning) trial (T1), two objects were placed in a symmetrical position at a distance of about 10 cm from the wall of the left- and the right-side of the arena. In the second (test) trial (T2), one object was moved to a new location which was about 20 cm forward or backward from the original position. Four different sets of objects were used. The different objects were: (1) a cone consisting of a gray polyvinyl chloride base (maximal diameter 18 cm) with a collar on top made of aluminum (total height 16 cm), (2) a standard 1 L brown glass bottle (diameter 10 cm, height 22 cm) filled with water, (3) a massive metal cube (10.0 cm × 5.0 cm × 7.5 cm) with two holes (diameter 1.9 cm), and (4) a solid aluminum cube with a tapering top (13.0 cm × 8.0 cm × 8.0 cm). Rats were unable to displace the objects.

Exploration was defined as follows: directing the nose to the object at a distance of no more than 2 cm and/or touching the object with the nose. Sitting on the object was not considered as exploratory behavior. A minimal amount of object interaction is required in order to achieve reliable object discrimination; therefore, rats that explore less than 7 s in T1 and/or 9 s in T2 were excluded from the analyses [46]. In order to avoid the presence of olfactory cues, the objects were always thoroughly cleaned after each trial with a 70% ethanol solution. All objects as well as the locations (left or right) of the objects were used in a balanced manner to avoid potential biases due to preferences for particular locations or objects.

In several studies it was shown that Wistar rats show a good object-location memory performance when a 1 h delay is interposed between the first trial and the second trial. However, when a 24 h delay is used rats do not discriminate between the novel and the familiar object-location in the second trial, indicating that the rats do not remember the object-location that was presented in the first trial. Using a 6 h delay, the discrimination performance is between that of the 1 h and 24 h delays, suggesting a delay-dependent forgetting in this task.

#### Procedure and Data Analysis

In the first two weeks, the animals were handled daily and were allowed to become accustomed to the test setup in two days, i.e., they were allowed to explore the apparatus (without any objects) twice for 5 min each day, after which they were allowed to explore the apparatus including the objects for 5 min on the next two days. Then, the rats were adapted to the testing routine until they showed a stable discrimination performance at the 1 h inter-trial interval (performance should be high) and the 24 h inter-trial interval (performance should be low). After this, an experiment was performed in which vericiguat was tested. All conditions were tested in 16 animals (except the vehicle condition, which was tested in 24 animals). Compounds were always administered 30 min before T1 to investigate the effects on the memory acquisition process. A 24 h inter-trial interval between T1 and T2 was used. The experimenter was always blinded to the conditions that were being tested. During testing the rats were assigned to treatment conditions in a balanced and randomized manner, thereby ensuring that all object combinations were distributed equally over the treatment conditions.

The basic measures were the times spent by rats in exploring an object during T1 and T2. The time spent in exploring the two symmetrically placed objects in T1 was represented by ‘a1’ and ‘a2’. The time spent in T2 in exploring the familiar and the novel object-location was represented by ‘a3’ and ‘b’, respectively. The following variables were calculated: e1 = a1 + a2, e2 = a3 + b and d2 = (b − a3)/e2 (see Table 1). e1 and e2 are measures of the total exploration time of both objects during T1 and T2, respectively. d2 is a relative measure of discrimination corrected for exploratory activity in the test-trial (e2). Thus, even if a treatment would affect exploratory behavior, the d2 index will be comparable between conditions. One-sample t-statistics were performed in order to assess per treatment condition whether d2 differed from zero. However, comparison of the mean d2 value with the value zero may not be the most suitable way to analyze recognition (increased chance of making a type I error). Therefore, results were also assessed using one-way ANOVA to compare with vehicle treatment. In the case of a significant difference between treatment conditions, pairwise post-hoc comparisons were performed using LSD *t*-tests.

### 2.5. cLTP Protocol, Surface Protein Biotinylation and Sample Preparation

Animals were sacrificed by means of cervical dislocation, the brains were excised and both hippocampi were isolated. Coronal hippocampal slices of 400 µm thickness were obtained using a McIlwain tissue chopper. The slices were transferred to chambers where the physiological conditions in the brain were maintained by artificial CSF (ACSF) continuously bubbled with 95% O_2_ and 5% CO_2_ at 37 °C. The ACSF composition was the following: 124 mM NaCl, 4.4 mM KCl, 1 mM Na_2_HPO_4_, 25 mM NaHCO_3_, 2 mM CaCl_2_, 2 mM MgSO_4_ and 10 mM glucose. Hippocampal slices were allowed to recover in ACSF under constant oxygenation bubbling at 37 °C for at least 2 h. Of note, for each animal the slices were used for all experimental conditions. Following the recovery, slices were transferred to a 6-well plate with each well designed as an individual incubation chamber in order to achieve simultaneous incubation for all experimental conditions. The order of the protocol was as follows: 10 min of incubation with vericiguat or vehicle (0.1% DMSO), 10 min rest in ACSF, 15 min of chemical LTP (cLTP) induction or vehicle (0.3% DMSO), and 15 min rest in ACSF before further processing and collection of the slices. The slices were under constant bubbling with O_2_ for the entire protocol. Vericiguat was incubated prior to the cLTP protocol to mimic the enhancement of acquisition processes in a memory paradigm (e.g., administration 30 min prior to T1 of the OLT).

The vericiguat stock was dissolved in DMSO and further diluted in ACSF immediately before use (end concentration of DMSO was 0.1%). The slices were either incubated with vehicle (0.1% DMSO) or vericiguat, which after a concentration–response pilot was determined to have an optimum concentration of 10 nM. Vericiguat was not present during the cLTP induction. For the induction of cLTP, stock solutions of forskolin and rolipram were dissolved initially in DMSO and thereafter in ACSF in order to reach a final concentration of 50 µM forskolin and 0.1 µM rolipram (end concentration of DMSO is 0.3%). The vehicle condition consisted of 0.3% DMSO, while the combination of forskolin and rolipram was considered a weak stimulation. After cLTP, slices were allowed to rest in ACSF for 15 min and were subsequently collected. For the concentration–response pilot, three concentrations were tested in combination with weak stimulation: 1 nM, 10 nM, 100 nM to determine the optimum concentration for follow-up experiments. For the full experiment with the optimum concentration (10 nM) of vericiguat, the conditions tested are described in Table 2.

Total membrane and intracellular AMPA (GluA1) receptor fractions were assessed using a biotinylation/precipitation protocol and Western blotting. Following the above cLTP protocol and 15 min rest in ACSF, slices were transferred to ice-cold ACSF containing 1 mM sulfo-NHS-SS-biotin and were left to incubate for 60 min on ice. Following biotin incubation, slices were washed with ice-cold 100 mM glycine to remove the excess of biotin and snap-frozen in liquid nitrogen. Frozen hippocampal slices were mechanically dissociated in lysis buffer (1 mM EDTA, 1 mM EGTA, 1% glycerol, 0.1% triton and 1% IGEPAL CA-630 in PBS), containing protease and phosphatase inhibitors. Protein concentration was determined with a Lowry protein assay (Bio-Rad Laboratories, Veenendaal, The Netherlands). For the membrane fractions, protein lysates (30 µg) were incubated overnight with streptavidin-coated Dynabeads at 4 °C under constant rotation. Dynabeads containing surface biotinylated proteins were separated from cytosolic proteins by magnetic precipitation. Biotinylated proteins were eluted from streptavidin beads with 1 × SDS loading buffer (1 M Tris HCl, 75% glycerol, 6% SDS, 15% β-mercaptoethanol and 0.025% brome phenol blue in milliQ) at 100 °C for 5 min.

#### Western Blotting and Data Analysis

Surface protein fractions and their corresponding total protein fractions (7 µg total protein) were resolved in 10% SDS-PAGE and then transferred onto nitrocellulose membranes (Bio-Rad Laboratories, Veenendaal, The Netherlands). The membranes were blocked (50% Odyssey blocking buffer in PBS, Li-Cor, Lincoln, NE, USA) for 1 h at room temperature, followed by overnight incubation with the primary antibodies at 4 °C. The primary antibodies consisted of mouse anti-glutamate receptor 1 N-terminus (1:1000, MAB2263, Merck Millipore, Burlington, MA, USA), rabbit anti-glutamate receptor 1 (AMPA subtype) (phospho S845) antibody (1:1000, ab76321, abcam) and mouse anti-GAPDH (1:1,000,000, 10R-G109A, Fitzgerald Industries, Acton, MA, USA), as loading control. Membranes were subsequently incubated by secondary antibodies for 1 h at room temperature: goat anti-rabbit IRDye 800 (1:10.000, Li-Cor, Lincoln, NE, USA) and donkey anti-mouse IRDye 680 (1:10.000, Li-Cor, Lincoln, NE, USA). Membranes were visualized using the Odyssey Infrared Imaging System (Li-Cor, Lincoln, NE, USA) and protein bands were quantified using ImageJ (https://imagej.nih.gov/ij/ (accessed on November 2017)). Raw intensity measures were normalized to GAPDH to control for loading differences. Outliers were excluded based on a Dixon’s Q test for outliers. The data were analyzed by using one-way ANOVA followed up by pairwise post-hoc comparisons using LSD *t*-tests.

### 2.6. Pharmacokinetic Measurements of Vericiguat

Brain and plasma sampling for PK measurements was carried out in rats used for behavioral tests after oral application of vericiguat (n = 4 per condition). In brief: rats were orally administered with 0.03, 0.3 and 3 mg/kg vericiguat. After 30 min, animals were sacrificed for brain collection. Prior to sacrifice blood samples were collected from the saphenous vein in EDTA-coated tubes. Plasma was isolated and together with the whole brain stored at −80 degrees Celsius until further processing. Following work-up and protein precipitation, concentrations in plasma and brain were determined by high pressure liquid chromatography (HPLC)-tandem mass spectrometry (MS/MS) methods as described previously [39].

### 2.7. Blood Pressure Measurements of Vericiguat

Blood pressure and heart rate were monitored in freely moving conscious animals by radiotelemetry by using the telemetric system (DSI Data Science International, St. Paul, MN, USA). At least two weeks prior to registration, rats underwent surgery for the implantation of telemetric implants (TA11PA-C40, DSI). After full recovery and for measurements, the rats were kept single in separate cages and signals were registered via receivers (RA1010) placed below the cage and analyzed by acquisition software Dataquest A.R.T 2.1.

Measurements of 2 h baseline mean arterial blood pressure (MAP) were taken before oral bolus application of 0.3, 1 or 3 mg/kg vericiguat (via gavage) or vehicle. Blood pressure was measured for an additional 24 h after injection. Data were analyzed as % of baseline. Heart rate was also monitored during that time.

### 2.8. Measurements of Cerebral Blood Volume

Rat cerebral blood volume was measured using magnetic resonance imaging (MRI). All animals were imaged using a Bruker Biospec 70/30 USR 7T MRI utilizing ParaVision 6.0.1 (Bruker, Ettlingen, Germany). Animals were initially anesthetized with 2.5% isoflurane in oxygen-enriched medical air, then switched to 0.3% isoflurane in 1 L/min medical air with 0.4 L/min oxygen with dexdomitor. Dexdomitor was given as a 25 ug/kg bolus at 0.1 mg/mL followed by 100 ug/kg/h infusion at 1 mL/kg/h. The animals were secured in the MRI prone, head first, with a 2 cm surface coil placed on the head. Body temperature was maintained at 37 °C using a water-circulating heating pad placed over the body of the rats, and respiration and temperature were monitored throughout the course of the study (SA Instruments, Inc., Stony Brook, NY, USA).

A tail vein catheter was implanted for contrast agent infusion using ferumoxytol (Amag Pharmaceuticals, Cambridge, MA, USA). Ferumoxytol was administered at 10 mg/mL as a 25 mg/kg bolus followed by a 3.75 mg/kg/hour infusion at the start of the experiment. Animals were allowed to acclimate for one hour in the MRI prior to experiment start for physiological stabilization of the animal; anatomical images were acquired during this time.

After one hour of stabilization, T2*-weighted echo planar images (EPI) were acquired for twenty minutes as a baseline (study start). After 20 min, vehicle (PEG400) or vericiguat was administered subcutaneously, and images were continuously collected for another 40 min. The vehicle-only (PEG400) and different doses of vericiguat studies were performed in a separate rat cohort. A total of 10 animals were given vehicle only, and vericiguat was administered at 0.03 (n = 4), 0.1 (n = 7), 0.3 (n = 7) and 1 mg/kg (n = 5). At the end of the image collection, a blood sample was taken for plasma PK analysis.

As a positive control, a separate n = 2 cohort underwent the same imaging procedure but 9 mg/kg sildenafil in saline was administered intraperitoneally, and no PK was collected. Here, the saline challenge or sildenafil was administered in the same paradigm as the PEG400 and sildenafil animals; 20 min was used for baseline collection, saline was intraperitoneally injected, and data were continuously acquired for another 40 min. Unlike the vericiguat study in which the vehicle and the vericiguat data were acquired from different animals, the vehicle and sildenafil data were acquired in the same rats during the same experimental session, i.e., the sildenafil animals were imaged for 120 min rather than 60 min to complete the saline-only and sildenafil paradigms in the same animals during the same imaging session.

The EPI image parameters included the following: 16 coronal slices using a single-shot gradient echo EPI sequence, matrix size = 64 × 64, field of view = 3 × 3 cm^2^, slice thickness = 1.3 mm, repetition time = 4 s, echo time = 11 ms. Thus, each measurement of the entire volume with 16 slices took 4 s.

All images were analyzed in Stimulate [53] where a rectangular region of interest (ROI) was placed covering the brain while excluding any non-brain regions; the time course of the signal intensity in the ROI was recorded. The percent signal change relative to the 20 min baseline was reported and plotted for each animal in each dose group.

## 3. Results

### 3.1. The Effects of Vericiguat on Memory Acquisition in the OLT

A one-way ANOVA revealed no differences between exploration times in T1 (e1: F (6113) = 2.03; *p* = n.s.) but it did show differences between exploration times in T2 (e2: F (6113) = 4.59; *p* < 0.001). Post-hoc LSD t-tests revealed that in T2, the vehicle condition showed a significantly lower object exploration when compared to the 0.03 and 1.0 mg/kg vericiguat and the 1.0 mg/kg donepezil conditions. Moreover, 0.03 mg/kg vericiguat showed a significantly higher object exploration when compared to all other vericiguat conditions. Finally, 0.3 mg/kg vericiguat showed a significantly lower object exploration when compared to the 1.0 mg/kg vericiguat and 1.0 mg/kg donepezil conditions (See Supplemental Table S1 for the exploratory means + SEM).

**Figure 1:**
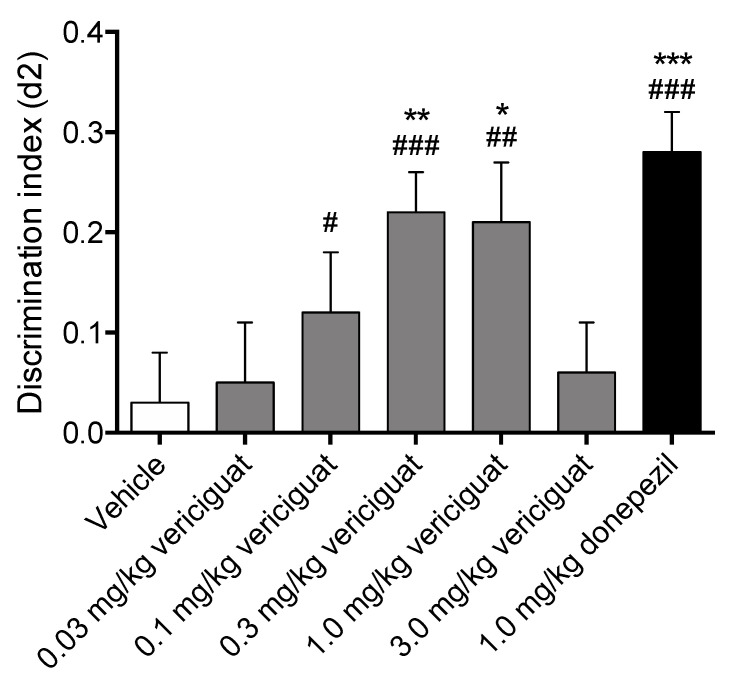
Dose–response curve of vericiguat on memory acquisition in the object location task. When compared with chance level (i.e., zero; d2 = 0), vericiguat administered 30 min before T1, improved memory performance at the doses of 0.1, 0.3 and 1.0 mg/kg. One-way ANOVA and subsequent post-hoc LSD t-tests revealed significant higher memory performance at 0.3 and 1.0 mg/kg vericiguat when compared to the vehicle condition. Reference compound donepezil was also administered 30 min before T1 and improved memory at 1.0 mg/kg, as indicated by both one-sample t-tests and one-way ANOVA and subsequent post-hoc LSD *t*-tests (comparison with vehicle). A difference from zero is depicted with hashes (One sample t-tests, #: *p* < 0.05; ##: *p* < 0.01; ###: *p* < 0.001) and a difference from the vehicle condition is depicted with asterisks (One-way ANOVA, LSD *t*-tests, *: *p* < 0.05; **: *p* < 0.01; ***: *p* < 0.001). The data are represented as mean + SEM.

Memory performance was significantly higher than zero for animals that received 0.1, 0.3 and 1.0 mg/kg vericiguat or 1.0 mg/kg donepezil, as indicated by the one-sample t-tests. No significant differences from zero were detected in the other treatment conditions (Figure 1). One-way ANOVA also showed significant differences in d2 indices between treatment conditions (d2: F (6, 113) = 3.83; *p* < 0.01). Post-hoc LSD t-tests revealed that when compared to the vehicle condition, both 0.3 and 1.0 mg/kg vericiguat showed significant higher memory performance. Furthermore, the reference compound donepezil also showed a significantly higher memory performance when compared to the vehicle condition. This indicates that 0.3 and 1.0 mg/kg vericiguat, and 1.0 mg/kg donepezil had a full effect on delay-dependent forgetting, whereas 0.1 mg/kg vericiguat only showed an intermediate effect on memory acquisition (being only significant from zero and not from vehicle).

### 3.2. Brain-to-Plamsa Ratio of Vericiguat

After oral administration of 0.03, 0.3 and 3 mg/kg to rats, concentrations of vericiguat were determined in plasma and brain after 30 min post dosing. Exposure in plasma and brain increased with dose and the obtained brain-to-plasma ratio (C_b_:C_p_) was at all doses ~0.01, indicating minimal brain penetration of vericiguat (Table 3).

### 3.3. The Effect of Vericiguat on AMPA Receptor Dynamics in an Acquisition-Like cLTP Model

**Figure 2:**
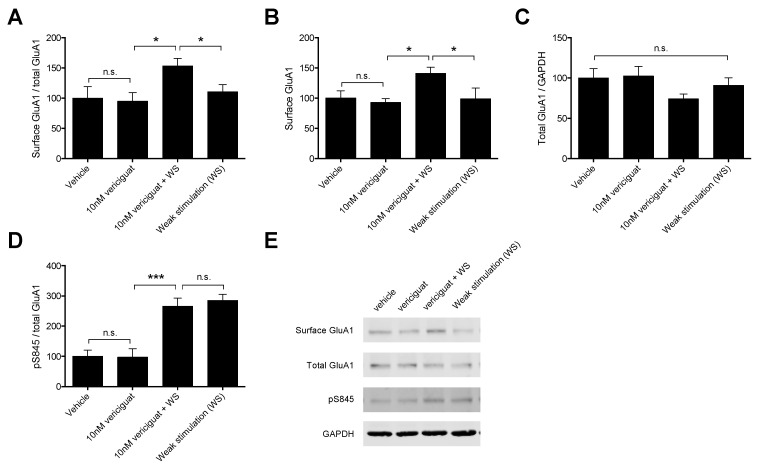
The effects of 10 nM vericiguat on AMPA receptor dynamics in acquisition-like processes in a chemical LTP model. (**A**) A combination of vericiguat with weak stimulation significantly enhanced mobilization of GluA1-containing AMPA receptors (AMPARs) to the surface, which cannot be attributed to effects of the drug alone. (**B**) The increase in surface GluA1-containing AMPAR protein levels together with (**C**) no effects on production of GluA1 suggests that increased trafficking is due to insertion of a pre-existing cytosolic pool of GluA1-containing AMPARs into the plasma membrane. (**D**) This observed increase in membrane insertion of GluA1 seems to be independent of S845 phosphorylation on the carboxy tail of GluA1, since vericiguat did not further enhance the increased pS845 by weak stimulation. (**E**) Representative Western blot for the AMPAR measures. Full Western blot images can be found in Supplemental Figure S1. Data are represented as mean + SEM. Differences between groups were analyzed with a one-way ANOVA and post-hoc LSD tests. * *p* < 0.05; *** *p* < 0.001; n = 9–11.

The optimum concentration of vericiguat in an acquisition-like cLTP model was established to be 10 nM in a preliminary concentration finding experiment (data not shown). Administration of 10 nM vericiguat in combination with weak stimulation (WS: a chemical stimulus induced with 50 µM forskolin and 0.1 µM rolipram to mimic a weak memory stimulus) significantly increased the mobilization of GluA1-containing AMPA receptors to the cell surface (as reflected by the surface GluA1/total GluA1 ratio) compared to weak stimulation alone (F(3, 32) = 3.014; *p* < 0.05 and post-hoc LSD: *p* < 0.05). This effect cannot be attributed to vericiguat alone since no effects were found for vericiguat without weak stimulation on AMPAR mobilization compared to vehicle. In addition, combining vericiguat with weak stimulation resulted in a significant increase in AMPAR mobilization compared to vericiguat alone (*p* < 0.05), indicating that the combination of a weak cLTP and vericiguat is necessary for enhancing GluA1-containing AMPAR mobilization to the surface (Figure 2A). Importantly, the observed increase in GluA1-containing AMPAR mobilization was due to an increase in surface insertion of an already pre-existing cytosolic (endosomal) pool of AMPAR, since vericiguat treatment showed the exact same effects on protein levels of surface GluA1-containing AMPAR as for the mobilization (F(3, 34) = 2.905; *p* < 0.05; Figure 2B), while no difference was found in the total number of AMPAR (F(3, 32) = 1.455; n.s.; Figure 2C). Additionally, phosphorylation of GluA1 on the serine 845 residue (pS845) was significantly enhanced by weak stimulation (F(3, 32) = 17.43; *p* < 0.001 and post-hoc LSD with vehicle: *p* < 0.001). However, this was not further enhanced by treatment with vericiguat (Figure 2D). Thus, the observed increase in GluA1-containing AMPAR insertion in the plasma membrane by vericiguat cannot be explained by increased phosphorylation at the S845 site on the GluA1 carboxy-tail.

### 3.4. The Effects of Vericiguat on Blood Pressure

**Figure 3:**
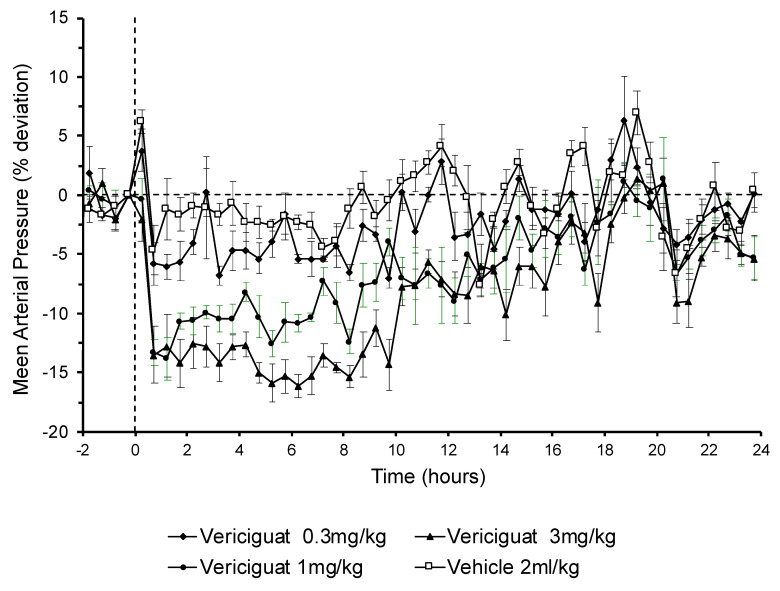
The effects of vericiguat on mean arterial blood pressure. Vericiguat induced a long-lasting drop in mean arterial blood pressure (MAP, % from baseline) after application of 1 or 3 mg/kg vericiguat (bolus p.o.). Data are represented as mean ± SEM (n = 4–5 in each group).

Blood pressure and heart rate were monitored in normotensive rats with telemetric implants. Vericiguat caused a dose-related and long-lasting decrease in mean arterial blood pressure (Figure 3). Oral administration of 0.3 mg/kg vericiguat had only a negligible effect on blood pressure. The blood pressure decreases started at 1.0–3.0 mg/kg; both lowered blood pressure by approximately 15%. Peak levels were reached within the first hour after administration. While the peak effect after 1.0 mg/kg started to fade out within the second hour, the peak effect after 3.0 mg/kg plateaued for more than 10 more hours. Dose-related transient tachycardia up to 20% with increasing doses was noted after all doses (data not shown).

### 3.5. The Effects of Vericiguat on Cerebral Blood Volume

**Figure 4:**
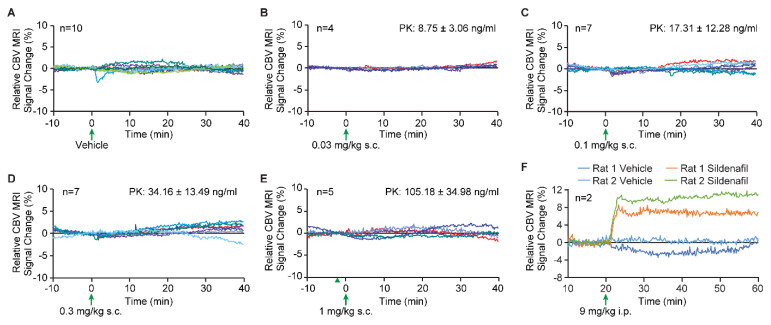
The effect of vericiguat for cerebral blood volume. No changes in cerebral blood volume were detected for any dose of vericiguat compared to vehicle injection. Plasma PK results for each dose group indicate successful delivery of vericiguat in a dose-dependent fashion. (**A**) Vehicle-only administration, (**B**) 0.03 mg/kg, (**C**) 0.1 mg/kg, (**D**) 0.3 mg/kg, (**E**) 1 mg/kg, and (**F**) 9 mg/kg sildenafil. Only the first 10 min of baseline and 40 min post-vehicle or vericiguat/sildenafil are shown for brevity, and each line for the vehicle and vericiguat plots (**A**–**E**) represents one animal. In (**F**), the vehicle and sildenafil data are from same rats.

Figure 4 summarizes the cerebral blood volume findings, where, as expected, PEG400 vehicle administration induces no changes in cerebral blood volume as detected by MRI. No differences between any tested dose of vericiguat demonstrated a change from the vehicle experiment, as well as from the 20 min of baseline imaging. However, to demonstrate that the MRI assay can detect an increase in cerebral blood volume, a 9 mg/kg dose of sildenafil administered intraperitoneally demonstrates a substantial increase in cerebral blood volume.

## 4. Discussion

In this study, the effects of vericiguat on long-term memory acquisition processes were measured in vivo by means of an OLT. Since the PK measurements indicated little to no brain penetration for vericiguat, the effects of vericiguat were also measured ex vivo by means of an acquisition-like cLTP model to determine the hippocampal plasticity effects in the absence of BBB limitations. To determine the effects of memory acquisition in vivo, vericiguat was administered orally 30 min before T1 at doses of 0.03–3.0 mg/kg in a 24 h interval paradigm. The AChE inhibitor donepezil was included as a reference compound and tested at the dose of 1.0 mg/kg. To determine the effects of vericiguat on acquisition-like processes ex vivo, a chemical LTP model was used and after a concentration-determining experiment (1, 10 and 100 nM vericiguat), vericiguat was tested at 10 nM to determine its effects on GluA1-containing AMPA receptor dynamics.

### 4.1. The Effects of Vericiguat on Memory Acquisition In Vivo in the OLT

An effect on exploratory behavior in the T2 was found for vericiguat and donepezil compared to vehicle. However, it is unlikely that the drugs interfered with the T2 since this was performed 24 h after the administration of the test compounds. Importantly, despite the statistical significant differences in exploratory behavior in T2, the mean exploration times of the animals were always sufficient to draw reliable conclusions [46]. Sporadic changes in exploratory behavior can occur during behavioral testing, and often the exact reasons remain unknown. Therefore, we interpret any differences in the amount of exploratory behavior to be incidental.

Vericiguat dose-dependently increased memory acquisition, showing a full effect on delay-dependent forgetting for 0.3 and 1.0 mg/kg, and an intermediate effect on delay-dependent forgetting for 0.1 mg/kg, while both the lowest dose of 0.03 mg/kg and the highest dose of 3 mg/kg did not improve memory performance. Additionally, 1.0 mg/kg donepezil as a reference compound for memory enhancement also showed a full effect on delay-dependent forgetting. A compound is considered to have a full effect on memory when the discrimination index is found to be significantly higher than both zero and vehicle in a 24 h interval paradigm, whereas an intermediate effect is considered to be only significant from zero [47]. Altogether this indicates that vericiguat is able to successfully enhance memory acquisition processes at 0.3 mg/kg and 1.0 mg/kg p.o. This is in line with previous research showing that PDE5 inhibitors, PDE9 inhibitors, and sGC stimulators/activators can enhance memory processes [21,28,54,55].

### 4.2. Brain Penetration Properties of Vericiguat

The plasma and brain exposure data after vericiguat treatment suggested that there is only minimal (concentration of vericiguat in brain only ~1% of those in plasma) and thus no relevant penetration of vericiguat into the brain. The C_b_:C_p_ ratio of vericiguat was 0.01 in this experiment, while the ratio of cerebral blood volume relative to total unperfused brain volume is close to 0.04, i.e., C_b_:C_p_ < 0.04 can be attributed to the vericiguat concentration in cerebral blood vessels [56]. This suggests that cognitive improvement may not be mediated by a central mode of action, since the BBB is limiting vericiguat’s in vivo brain penetration, and memory enhancement is most likely through a peripheral mechanism.

### 4.3. The Effects of Vericiguat on Acquisition-Like Processes in an Ex Vivo cLTP Model

Initially, a dose–response curve of vericiguat was established to determine the optimum concentration of vericiguat for the full cLTP experiments. Three doses of vericiguat were tested (1, 10 and 100 nM) in combination with weak stimulation to determine the effects on GluA1-containing AMPA receptor mobilization to the surface as reflected by the surface GluA1/total GluA1 ratio in Western blot. Experiments with PDE5 inhibitor vardenafil and PDE4 inhibitor rolipram on memory acquisition have shown that during the acquisition process, it is most likely to find an effect on GluA1 mobilization, whereas drug administration during consolidation processes seem to enhance GluA1 production [21]. Therefore, we focused specifically on GluA1 mobilization for the concentration–response experiments. A quantity of 10 nM vericiguat was chosen as the optimal concentration for the follow-up experiments, as this concentration showed the highest absolute increase in GluA1 mobilization to the surface compared to weak stimulation alone.

For the follow-up experiments, a vehicle and vericiguat-only condition were added to gain a better understanding of vericiguat-only effects (i.e., without weak stimulation) on AMPAR dynamics in addition to the effects of vericiguat in combination with weak stimulation. Vericiguat was found to enhance mobilization of GluA1-containing AMPARs to the surface when combined with weak stimulation, but not by itself, suggesting that an LTP stimulus is needed for this effect. In addition, the increased mobilization was found to result from the increased insertion of a pre-existing pool of GluA1-containing AMPARs, since surface GluA1 protein levels were affected, but total GluA1 levels were not. This is in line with the previously mentioned experiments by Argyrousi et al. [21], who found similar results for vardenafil during memory acquisition processes in an in vivo setting.

There are multiple ways through which the sGC-cGMP-PKG pathway can affect GluA1 mobilization to the surface, of which phosphorylation at S845 is thought to promote both targeting and retention of GluA1 at the post-synaptic density (PSD) (for an extensive review see [26]). Therefore, we measured whether the increased GluA1 mobilization induced by vericiguat could result from increased S845 phosphorylation. Our results show that weak stimulation itself enhanced pS845 compared to vehicle, which is expected since WS was induced by enhancing the cAMP-PKA pathway that is known to phosphorylate S845 as well as PKG, and these results are in line with previous research using the cLTP method [57]. However, combining vericiguat with WS did not enhance pS845 phosphorylation compared to weak stimulation alone. This result is unexpected, since WS itself did not enhance GuA1 mobilization to the surface whereas the combination of vericiguat and WS did. It was previously found that increased phosphorylation of S845 leads to an increase in GluA1-containing AMPA receptors in the cell membrane [25,57,58,59]. However, it has also been suggested that phosphorylation on S845 is not sufficient for increased insertion of GluA1 into the membrane and might require additional CaMKII activity [60]. Moreover, it is thought that S845 phosphorylation results in membrane insertion of GluA1 but does not lead to recruitment into the PSD [61], meaning that the lack of increased S845 phosphorylation after vericiguat treatment when combined with WS could even be beneficial for the recruitment of GluA1 to the PSD rather than perisynaptically. Indeed, our quantification method does not allow us to distinguish between membrane locations (i.e., PSD, perisynaptically, extrasynaptically). Nonetheless, it can be concluded that the observed increase in GluA1 insertion into the membrane induced by vericiguat likely does not rely on S845 phosphorylation and may involve other intracellular mechanisms.

However, based on our PK measurements, vericiguat is unable to cross the BBB. Experiments with riociguat, another sGC stimulator incapable of penetrating the brain, indicated that there were no in vivo effects on hippocampal plasticity measures including GluA1-containing AMPARs [36]. Therefore, the ex vivo experiments with vericiguat were conducted to investigate whether vericiguat has the potential to enhance hippocampal plasticity via GluA1-containing AMPARs in the absence of the BBB limitations. Under healthy conditions, the BBB is not permeable to many pharmaceuticals, which includes vericiguat. However, specifically in VCI the BBB integrity is compromised and the BBB is considered “leaky” [2,62]. Under such pathological conditions, the BBB may no longer be a limiting factor for vericiguat and hypothetically, vericiguat may then be able to enhance neuronal plasticity and aid in the treatment of VCI.

### 4.4. The Effects of Vericiguat on Blood Pressure

Vericiguat and other sGC stimulators can dose-dependently reduce blood pressure. Since the long-term memory showed an inverted u-shaped dose–response relationship, it could not be ruled out that a decrease in blood pressure in higher dosages might be involved in the absence of memory-enhancing effects. In fact, blood pressure monitoring in conscious rats showed that 3 mg/kg of oral vericiguat, which had no significant effect on memory improvement, had a long-lasting blood pressure lowering effect which could be observed for at least 10 h after application. In addition, the 1 mg/kg vericiguat dose had a similar initial blood pressure lowering effect. However, this was less pronounced over time compared to the 3 mg/kg dose. The observed increase in MAP reduction with dose is in line with the observed increased exposures of vericiguat with higher doses. The 0.3 mg/kg vericiguat dose was highly effective on memory function but had no effect on blood pressure. Additionally, the highest dose tested in the object location task of 3 mg/kg vericiguat did not improve memory performance while 1 mg/kg was still found to be effective. Since 3 mg/kg vericiguat showed the most pronounced effect on blood pressure, it can be argued that the hypotensive effects of vericiguat at doses above 1 mg/kg are detrimental for memory or even counteract mechanisms important for memory enhancement. Overall, these data are consistent with the hypothesis that long-lasting and pronounced blood pressure lowering effects could actually be responsible for a decline in cognitive function. Moreover, these data suggest that vericiguat improves cognitive function in a dose range which has no or moderate blood pressure lowering effects.

### 4.5. The Effects of Vericiguat on Cerebral Blood Volume

It is well established that sGC stimulators dilate a broad variety of blood vessels. If sGC stimulators can also dilate vasculature in brain, the vascular resistance would be decreased, and this decrease in vascular resistance would cause an increase in cerebral blood flow. Additionally, it might be conceivable that improved cerebral blood flow contributes to enhanced memory performance. Since it is well established that cGMP and sGC stimulators are potent vasorelaxant molecules, one potential peripheral mechanism for enhancing memory with vericiguat could be an increase in cerebral blood flow. Here, the cerebral blood volume measurements obtained from MRI, which measures the vascular dilation in brain parenchyma, demonstrated no change in blood volume from baseline or vehicle treatment, and with different doses of vericiguat, thus no change in vascular dilation. To confirm the ability of the MRI assay to measure dilation-induced blood volume changes, a marked increase was observed when sildenafil was used as a positive control. In line with this, a previous study with the PDE5 inhibitor vardenafil found no hippocampal cerebrovascular effects (i.e., cerebral blood flow and glucose utilization) at a dose that improved spatial memory performance [63]. Vardenafil has been proven to be brain penetrant, thus suggesting a direct central effect on memory, e.g., by increasing neuronal signal transduction [64]. Yet even PDE5 inhibitors and sGC stimulators such as UK343,664 and riociguat, respectively, were able to enhance spatial memory in rodents despite their inability to penetrate the brain [36,64]. This suggests that a peripheral cGMP-related mechanism might underlie possible improvements in memory function. In the riociguat study there was an indication for a vascular component in the brain as the transcription factor VASP was activated [36]. However, in those studies no blood pressure or blood flow was assessed, yet the drugs’ doses were in the lower ranges, and thus the effect on blood flow is very likely little to none.

### 4.6. Alternative Effects

Instead of hemodynamic effects, it can also be speculated that sGC stimulation enhances energy metabolism by activating natriuretic peptides in the cardiovascular system, regulating mitochondrial biogenesis through cGMP signaling in muscle tissue, and increasing glucose metabolism [65,66,67]. Peripheral mechanisms likely also underly the memory enhancing effects in rodents for the likewise poorly brain penetrant sGC stimulator riociguat, which in a novel study by us was found to reverse the short-term memory impairments induced by cerebral vasoconstrictor sumatriptan with an altered dose–response curve, implying that riociguat may act through vasoactive mechanisms [36]. Unfortunately, a first exploratory clinical trial with riociguat failed [68], which was discussed as being probably related to its poor brain penetrance. However, it is likely that its very narrow therapeutic window on cognition could also play a role, i.e., the optimum dose for a (likely peripheral) effect was missed. Interestingly, in a recent study Correia et al. [69] compared the novel brain penetrant sGC stimulator CY6463 to vericiguat and discovered that vericiguat did not increase cGMP levels in the CSF of rats after oral dosing with 30 mg/kg. In addition, altered fMRI-BOLD signals were found in only peripheral cortical regions after intravenous infusion of 3 mg/kg. In contrast, CY6463 increased cGMP levels and induced an fMRI-BOLD signal change in hippocampal cortical and dopaminergic midbrain regions. Similar to our study, vericiguat reduced MAP, albeit in a 10x higher dose-range. The effective vericiguat doses in the study by Correia et al. [69] are significantly higher than our memory-enhancing effective dose of 0.3 mg/kg. However, the differences between the brain-penetrant sGC stimulator CY6463 and the non-penetrant sGC stimulator vericiguat suggest mechanistically different pathways in memory enhancement. Altogether the results from our study further emphasize the need for a better understanding of peripheral but also central cGMP-mediated mechanisms of memory enhancement to overcome translational discrepancies between animals and humans. This then helps us to explain the underlying mechanism(s) of an improvement in cognition, i.e., direct centrally, vascular peripherally, or non-vascular peripherally.

The current study focused mainly on male rodents for the memory experiments and hippocampal (GluA1-containing AMPAR) plasticity measurements. As mentioned previously, the study was designed as an explorative study into the memory enhancing potential of sGC stimulator vericiguat without expectations with regards to the effectiveness. Therefore, the most optimal sex was used for detecting effects within the OLT and cLTP techniques, i.e., male rodents. Previous research indicated that estrogen and the estrous cycle can both influence memory performance and weak tetanus responsiveness in rodents [45,70]. In addition, the NO-sGC-cGMP system also seems to be particularly affected by estrogen (e.g., see [71,72]). Therefore, future (preclinical) research with vericiguat should include female rodents to check for possible effects of the estrous cycle, which can further build on the effects of the current first-in-line explorative study using vericiguat as a cognitive enhancer.

### 4.7. Conclusions

Vericiguat enhances memory acquisition in vivo in an object location paradigm for spatial memory in rats. However, these effects are likely via a peripheral mechanism on the cerebral microvasculature, since vericiguat was unable to penetrate the brain. In an ex vivo model for memory acquisition processes, vericiguat enhanced the mobilization of GluA1 AMPARs to the surface and GluA1 insertion into the membrane. In this ex vivo model, the effects of vericiguat could not be hampered by the BBB, and the potential of vericiguat to enhance neuronal plasticity was shown. Despite vericiguat’s inability to penetrate the brain under physiological conditions, this may have important additional implications for the treatment of cognitive impairments under pathological conditions in which the BBB integrity is impaired, such as vascular cognitive impairments. Therefore, sGC may hold promise as a target for the treatment of VCI, yet more research is needed, ideally comparing both brain-penetrant and non-penetrant sGC stimulators.

## 5. Declaration of Interest

M.G., J.H., P.S. are employees of Bayer AG, Pharmaceuticals, and F.Z. was an employee of Merck Sharp & Dohme Corp., a subsidiary of Merck & Co., Inc., Kenilworth, while these studies were performed. C.H., G.S. are employees of Merck Sharp & Dohme Corp., a subsidiary of Merck & Co., Inc., Kenilworth, NJ, USA. E.N., E.K.A., N.P.v.G., J.P. were supported by a restricted research grant by Bayer AG and by Merck Sharp & Dohme Corp., a subsidiary of Merck & Co., Inc., Kenilworth, NJ, USA.

## Figures and Tables

**Table 1:** Measures involved in the object location test (OLT).

Exploration	Discrimination
e1 = a1 + a2	
e2 = a3 + b	d2 = (b − a3)/e2

e1 is the measure of the time spent in exploring both symmetrically placed objects (a1 and a2) in the first trial, and e2 is the measure of the time spent in exploring both the familiar (a3) and the new object-location (b) in the second trial; d2 corresponds to the ability to discriminate between the familiar and novel object-location during the second trial and is corrected for exploration time during that trial.

**Table 2:** Incubation conditions during the 10 nM vericiguat ex vivo cLTP experiment.

Experimental Condition	Treatment during 10 min Vericiguat Incubation	Treatment during cLTP Protocol
Vehicle	0.1% DMSO	0.3% DMSO
Vericiguat only (without WS)	10 nM vericiguat	0.3% DMSO
Vericiguat + WS	10 nM vericiguat	50 µM forskolin + 0.1 µM rolipram
Weak stimulation only	0.1% DMSO	50 µM forskolin + 0.1 µM rolipram

Described are the experimental conditions and corresponding exposures during the vericiguat incubation period and the chemical LTP protocol.

**Table 3:** Pharmacokinetics of vericiguat.

Vericiguat 0.03 mg/kg	
C_p_ (ng/mL)	25.75 (1.55)
C_b_ (ng/g)	0.33 (0.05)
C_b_:C_p_	0.01 (0.00)
Vericiguat 0.3 mg/kg	
C_p_ (ng/mL)	88.90 (11.76)
C_b_ (ng/g)	1.07 (0.16)
C_b_:C_p_	0.01 (0.00)
Vericiguat 3 mg/kg	
C_p_ (ng/mL)	1543.86 (325.00)
C_b_ (ng/g)	16.67 (4.03)
C_b_:C_p_	0.01 (0.00)

The brain concentration:plasma concentration ratio (C_b_:C_p_) of vericiguat was 0.01 for all three doses tested (0.03, 0.3 and 3 mg/kg bolus p.o.). Data are represented as mean (SEM).

## Data Availability

The data presented in this study are freely available on request from the corresponding author.

## Supplementary Materials

The following are available online at https://www.mdpi.com/article/10.3390/biomedicines9081047/s1, Supplementary Figure S1: full images of the western blot shown in Figure 2E, Supplemental Table S1. Total exploration ties of vericiguat and donepezil in the OLT.
