# Supplementary material for: Soluble Guanylate Cyclase Stimulator Vericiguat Enhances Long-Term Memory in Rats without Altering Cerebral Blood Volume
Source: Biomedicines. 2021 Aug 19;9(8):1047. doi: 10.3390/biomedicines9081047 (PMC8393324; doi:10.3390/biomedicines9081047)
Supplement: Supplementary file 1 [file biomedicines-09-01047-s001.zip › biomedicines-1326165-Supplementary.pdf]

Supplemental figures

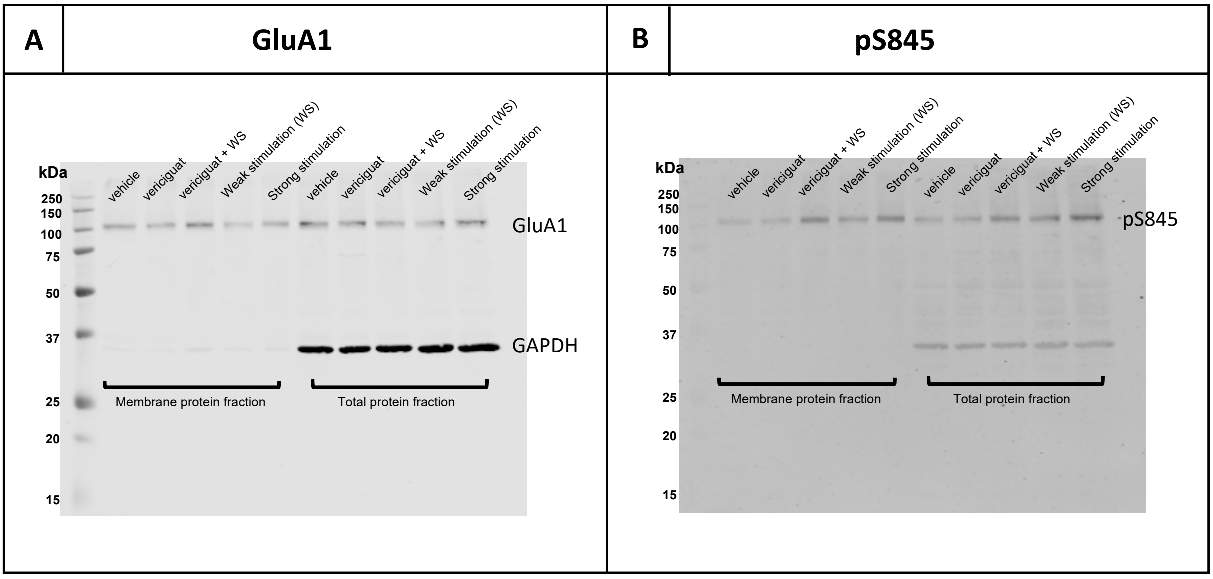

Supplementary figure S1: full images of the western blot shown in Figure 2E. A) GluA1 and GAPDH are mouse-derived antibodies and measured in the 700 channel of an Oddysey CLx scanner using an IRDye 680 secondary antibody. GluA1 is 110kDa and GAPDH is 36kDa. GAPDH is a cytosolic protein, thus a purified membrane protein fraction should not contain GAPDH. **B)** S845 phosphorylation of GluA1 is detected with a rabbit-derived antibody and measured in the 800 channel of an Oddysey CLx scanner using an IRDye 800 secondary antibody. Similar to GluA1, this can be found at 110kDa. Only the total protein fraction was analyzed for pS845. Of note, a strong stimulation condition can be found on the blots. The strong stimulation did not work and was therefore not taken into the final analysis.

Supplemental tables

Supplemental Table S1. Total exploration times of vericiguat and donepezil in the OLT

	Vehicle	0.03 mg/kg vericiguat	0.1 mg/kg vericiguat	0.3 mg/kg vericiguat	1.0 mg/kg vericiguat	3.0 mg/kg vericiguat	1.0 mg/kg donepezil
**e1**	43.81 (1.63)	52.45 (2.44)	44.95 (2.22)	41.98 (3.09)	43.18 (2.48)	47.05 (2.82)	42.84 (2.84)
**e2**	36.34 (1.54)	48.72 (1.98)	39.06 (1.98)	37.04 (2.26)	43.93 (2.00)	40.99 (2.74)	43.34 (2.11)

The different dosages of vericiguat and donepezil were administered 30 min before T1. The delay interval between the first and second trial was 24 h. n=24 for vehicle, n=16 for all other groups.
